# A study on β-defensin-2 and histatin-5 as a diagnostic marker of early childhood caries progression

**DOI:** 10.1186/s40659-015-0050-7

**Published:** 2015-10-31

**Authors:** Anna Jurczak, Dorota Kościelniak, Monika Papież, Palina Vyhouskaya, Wirginia Krzyściak

**Affiliations:** Department of Pediatric Dentistry, Institute of Dentistry, Jagiellonian University, Medical College, Krakow, Poland; Department of Cytobiology, Pharmacy Faculty, Jagiellonian University, Medical College, Krakow, Poland; Department of Medical Diagnostics, Pharmacy Faculty, Jagiellonian University, Medical College, 9 Medyczna St., 30-688 Krakow, Poland

**Keywords:** β-defensin-2, Histatin-5, Early childhood caries

## Abstract

**Background:**

Recently, a continuous growth of interest has been observed in antimicrobial peptides (AMPs) in the light of an alarming increase in resistance of bacteria and fungi against antibiotics. AMPs are used as biomarkers in diagnosis and monitoring of oral cavity pathologies. Therefore, the determination of specific protein profiles in children diagnosed with early childhood caries (ECC) might be a basis for effective screening tests and specialized examinations which may enable progression of disease.

**Methods:**

The objective of the studies was to determine the role of histatin-5 and β-defensing-2 as a diagnostic marker of early childhood caries progression. In this work, results of concentration determination of two salivary proteins (histatin-5 and β-defensin-2) were presented. In addition, bacterial profiles from dental plaque in various stages of ECC and control were marked. The assessment of alteration in the concentration of these two proteins in a study group of children with various stages of ECC and a control group consisting of children with no symptoms was performed by enzyme-linked immunosorbent assays.

**Results:**

The statistical analysis showed a significant increase in the concentration of histatin-5 and β-defensin-2 in the study group compared to the control group and correlated with the progression of the disease.

**Conclusions:**

The confirmation of concentration changes in these proteins during the progression of dental caries may discover valuable disease progression biomarkers.

## Background

Interest for saliva as a study material is a consequence of the search for body fluid obtaining of which is easy, non-invasive, and relatively inexpensive [1, 2]. It is also possible to determine salivary proteins occurring in small concentrations. Salivary peptides have been used in monitoring pathological conditions such as oral cavity, larynx and breast cancers [3], systemic sclerosis, Sjögren’s syndrome, cirrhosis, and mucoviscidosis [4]. Currently, saliva analysis is also used in detecting infectious diseases [5].

Histatins (HST) and defensins are antibacterial proteins of the oral cavity, and their biological activity is directed toward the protection against infectious diseases, including dental caries. The development of caries is a consequence of imbalance of a natural equilibrium ensured by inborn defense systems. Periodical physiological changes of concentrations of the mentioned proteins aim at restoring ecological balance in the oral cavity.

It seems that these proteins may be involved in preventing numerous diseases such as dental caries, and because these proteins naturally occur in the oral cavity they should be clinically well tolerated.

### Defensins

The role of defensins is still not yet entirely understood. Saliva contains both α-defensins and β-defensins. Alpha-defensins (HNP-1,2,3) are secreted by neutrophils, whereas β-defensins (HBD-1,2) undergo a specific expression in salivary duct cells, not in salivary gland vesicles. The increase of the number of neutrophils in blood affects an increase in α-defensin concentration in saliva [6]. A temporary, increased level of α-defensins is related to, i.e. acute inflammation, fever, poisoning, hemorrhage, quickly developing malignant tumor [7].

They have local effect—protect salivary glands against bacterial invasion (Gram-negative: *P. aeruginosa*, *E. coli*; Gram-positive: *S. aureus*, *S. pneumoniae*, *S. faecalis*), viruses (HIV-1, HSV-1, HAdV), and fungi (*C. albicans*).

β-Defensins in vitro exhibit cytotoxic activity against different eukaryotic and tumor cells [8–10]. In physiologic in vitro concentrations, HBD stimulate the proliferation of these cells, while in higher concentrations they are cytotoxic to them [11]. β-Defensins also participate in specific immune response as chemotactic factors—they adhere to CCR6 chemokine receptor on dendritic cells [12].

**Fig. 1:**
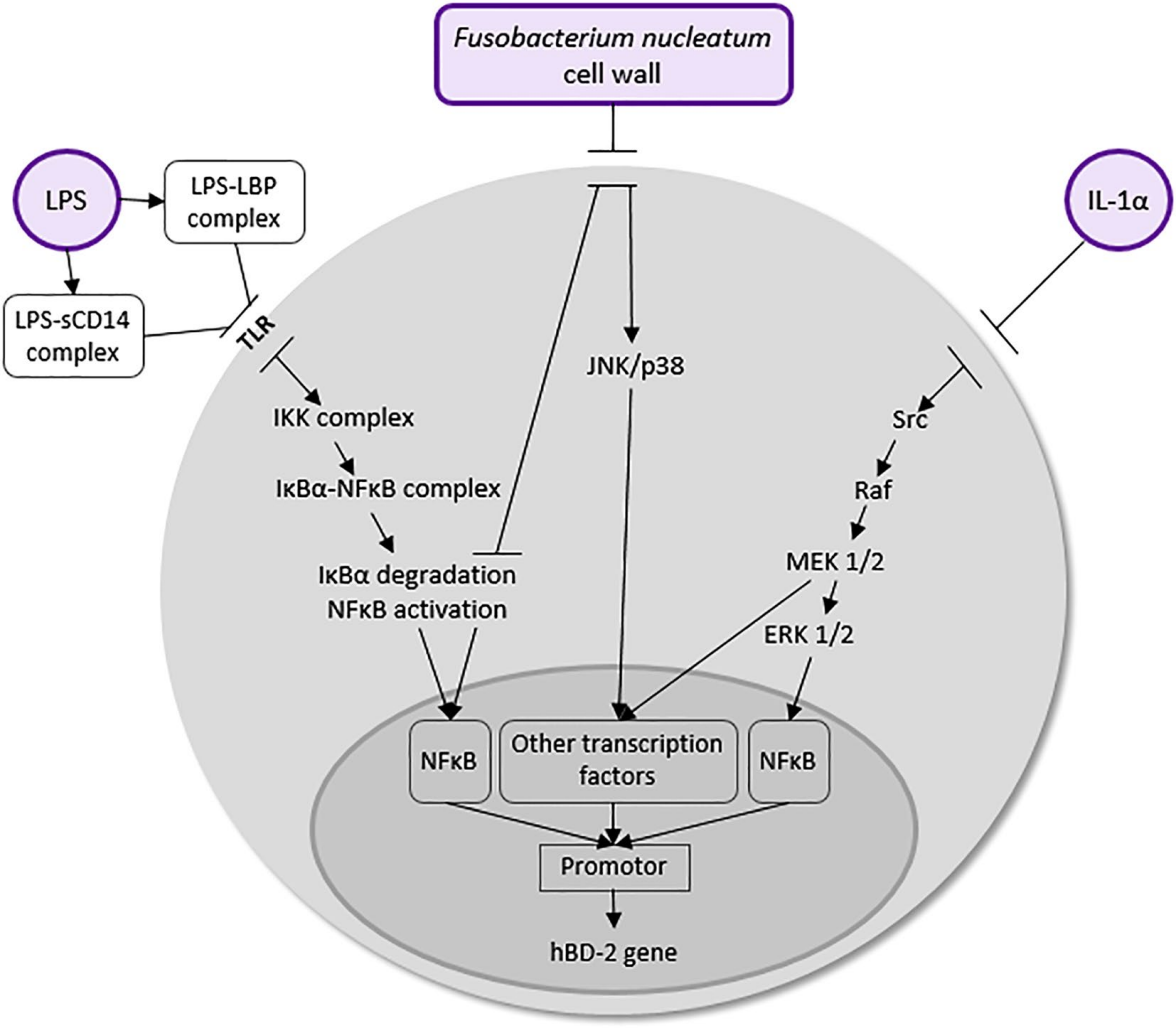
Signaling pathways in inducible expression of HBD-2 in epithelial cell. (*ERK* extracellular-signal-regulated kinase, *hBD-2* human β-defensin-2, *IκBα* nuclear factor of κ light polypeptide gene enhancer in B-cell inhibitor, alpha, *IKK* IκB kinase, *IL 1a* interleukin 1a, *JNK* c-Jun N-terminal kinase, *LBP* lipopolysaccharide binding protein, *LPS* lipopolysaccharide, *TLR* toll-like receptor, *MEK* mitogen-activated protein kinase, *NFκB* nuclear factor κ-light-chain-enhancer of activated B cells, *Raf* proto-oncogene serine/threonine-protein kinase, *sCD14* soluble CD14, *Src* proto-oncogene tyrosine-protein kinase Src)

The expression of β-defensin-2 (HBD-2) is induced by proinflammatory cytokines (IL-1β, IFN-γ, TNF-α) in complex signaling pathway [13–15] activated by the effect of LPS lipopolysaccharide on epithelial cells depending on mitogen-activated protein kinases (MAPK) (Fig. 1) [15].

### Histatins

Histatins (HST)—produced in parotid and sublingual salivary glands—are among the main natural antimicrobial proteins of saliva. Among the 12 main forms of proteins occurring in saliva are HST-1, -3 and -5. HST-1 and -3 are the products of different genes, and HST-2 is formed as an effect of post-translational HST-1 modification. The remaining types are formed from HST-3 transitions or HST-5 and -6 degradation. HST-1, -3, and -5 constitute 85 % of the total content of these proteins in saliva, and HST-3 and -5 have the strongest antimicrobial activity. HST-1, -3, and -5 also constitute components of the *acquired enamel pellicle* (AEP).

HST destabilize cellular membrane of bacteria by assimilating with its surface leading to cell damage. Moreover, HST decrease outflow of proinflammatory cytokines (interleukins, TNFα, metabolites of arachidonic and other acids) as a response of organism to stimulation of cell walls of Gram-negative bacteria with lipopolysaccharide [16]. A range of antibacterial effect includes microbes such as *S. mutans*, *S. aureus*, *P. gingivalis*, *E. coli*, and others [17, 18].

**Fig. 2:**
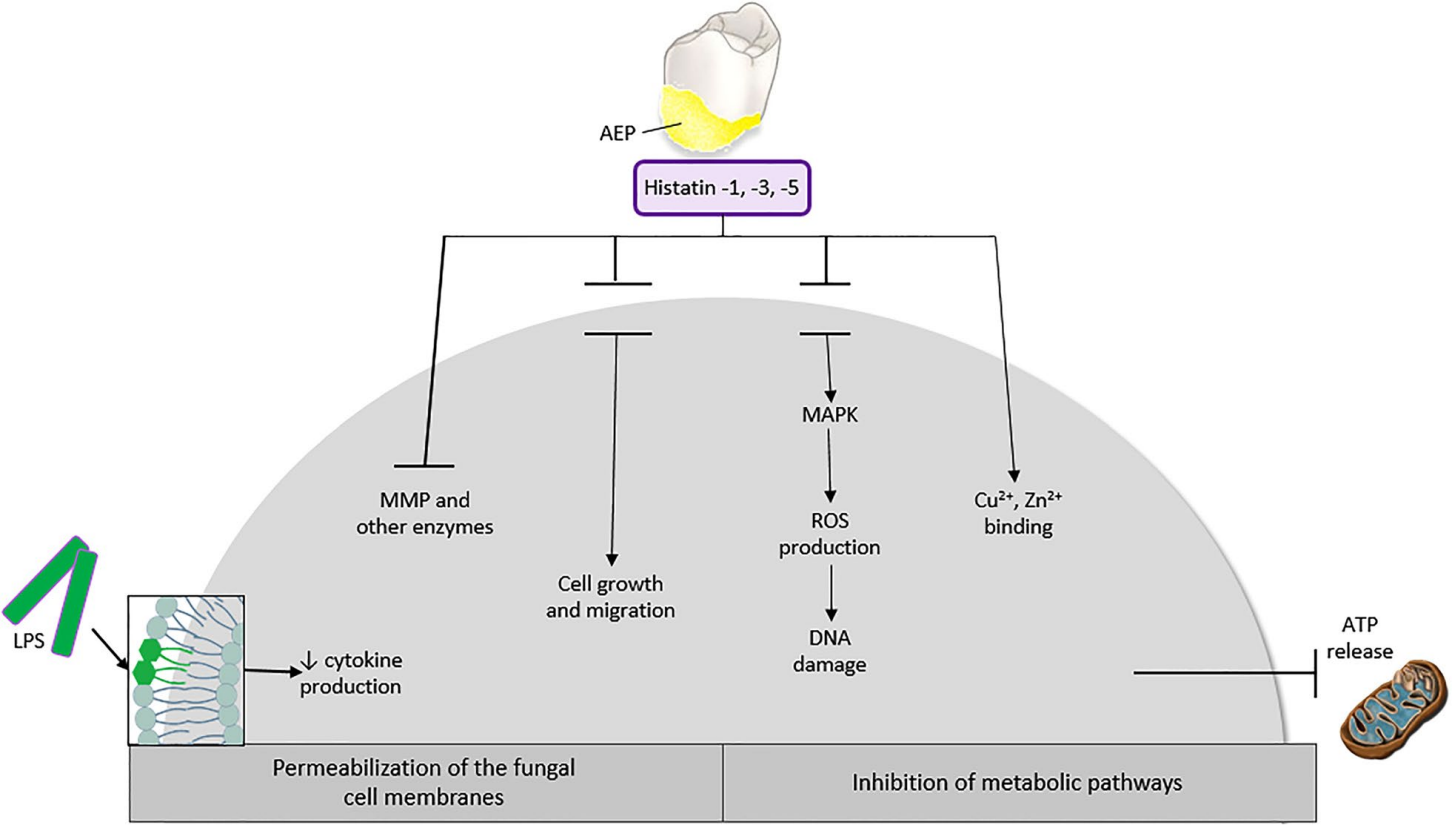
Signaling pathways in inducible expression of HST in epithelial cell. (*AEP* acquired enamel pellicle, *LPS* lipopolysaccharide, *MAPK* mitogen-activated protein kinase, *MMP* matrix metalloproteinases, *ROS* reactive oxygen species) [24]. In the mechanism depending on protein kinases (MAPK), HST specifically assimilate with membrane receptors via a series of mechanisms inducing the production of reactive oxygen species (ROS) [25]. HST by a way of non-lytic outflow from cell lead to the release of ATP and inhibition of the main metabolic pathways (i.e. cellular respiration) [26]. Together with reactive oxygen species, HST release histamine from mast cells [27] and lead to DNA damage [28]. HST have a function of chelating transition metal ions (Cu^2+^ and Zn^2+^), which may be of importance for the protection of tooth enamel and antimicrobial protection

HST-5 was demonstrated as the only protein from the HST group exhibiting antiviral activity (against HIV-1—inhibition of replication by the active domain of HST-5—Dh-5) [19, 20] (Fig. 2).

Among other HST functions known is their activity as inhibitors of proteolytic enzymes originating from microbes (metalloproteases, trypsin-like enzymes, cysteine proteases) or host (collagenases) [18, 21]. Especially important is the inhibition of collagenases (MMP) activity of which increases under pathological conditions and is related to inflammatory and degenerative diseases and carcinogenic processes. Currently, HST are considered to be one of the dominant substances of saliva participating in wound healing [22]. HST acting synergistically with epidermal growth factor (EGF) activate G protein-coupled receptors, which in consequence lead to growth and migration of cells (Fig. 2) [23].

The objective of this study was an attempt to use the determination of salivary proteins: HST-5 and β-defensin-2 as markers of dental caries progression at various stages of its development.

## Methods

### Subject of the study

The study was conducted between 2010 and 2013 and included 82 pediatric patients from the Department of Pediatric Dentistry, Institute of Dentistry, Jagiellonian University, Medical College in Krakow, Lesser Poland Małopolska province. The study included strains isolated from patients (*n* = 41; average age = 5 ± 2.3) diagnosed with ECC in deciduous teeth. Patients were divided in two groups: the mild group (1–2 in ICDAS II codes; n = 17) with the initial demineralization involvement (white spots/non-cavitated lesion) and severe group (with cavitated lesion) which was considered to be ≥3 in ICDAS II codes (*n* = 24). The control group was formed of strains isolated from children (*n* = 41; average age = 5 ± 1.5) without caries lesions in deciduous teeth, who were kept under the clinic’s control (Table 1). In a clinical evaluation of the patients, the presence of tooth decay in deciduous and permanent teeth, number of teeth removed due to caries, and filled teeth were assessed. The study describing the indexes of caries, dmf/DMF and the frequency, was conducted in accordance with the criteria established by the World Health Organization (1997) for epidemiological studies (Oral Health Surveys Basic Data) in artificial lighting using a dental mirror and probe.

**Table 1:** Salivary HST-5 and β-defensin-2 levels in early childhood caries and controls

	Healthy control (*n* = 41)		Early childhood caries (*n* = 41)		p*
	Mean (ng/ml)	SD	Mean (ng/ml)	SD	
HST-5	15.29	1.16	50.75	2.11	0.0002
β-defensin-2	2.15	0.07	2.29	0.05	0.0417

* *t* Test was used in the analysis

The classification of patients for the study was performed during a routine dental examination. This examination was conducted by a qualified dentist (internal examiner), with many years of experience in epidemiological studies of the oral cavity. Kappa coefficient was set at 0.91 in the pilot studies, which indicates the internal examiner’s reliability. The legal guardians of the participants of the study were fully informed about its aim and course and agreed on their children’s participation. The factors excluding from participation in the study were insufficiently developed chewing activity observed in small children and mentally disabled patients, and the lack of an agreement from legal guardians for their children’s participation in the experiment.

### Material collection

Saliva and plaque samples were obtained from children with caries and also from children with no caries symptoms. After the classification of the patients for the research, scrapings from carious foci (in the case of children with caries) or from dental plaque (in children without caries) were collected for the examination using an open system composed of a sterile cotton swab placed in a test tube. Unstimulated saliva samples (2 ml) were collected into sterile plastic tubes in the morning before tooth-brushing and any clinical examination. Saliva samples were homogenized and clarified by centrifugation at 10,000×*g* for 15 min at 4 °C. The aliquots of clarified supernatants were kept at −70 °C until needed for the measurements.

The patients maintained a fasting status, and sampling was performed between 8 and 10 am after the prior oral cavity rinsing with distilled water. The collected material was sealed in sterile test tubes, inhibiting oxygen access, and transported at room temperature to the laboratory within a time period of no longer than 1 h. Saliva samples were centrifuged at 1500×*g* for 10 min; supernatants were collected and stored at −20 °C. Then, salivary β-defensin-2 and HST-5 levels were measured by ELISA (BIO-RAD, Hercules, CA, USA).

### Bacterial culture of collected material on HLR-S medium

The material was inoculated on a selective medium (HLR-S) in the laboratory. The range of inhibition of the growth of bacteria colonizing an oral cavity was verified in a preliminary study on four media, which are described in the literature as selective for this kind of pathogens [29, 30]. The results are presented in our previous manuscript [28].

### Bacterial identification

Enzymatic typing was performed based on the enzymatic profiles obtained from the STREPTOtest 24 test (Lachema, Pliva) described in our previous manuscript [28].

### Salivary β-defensin-2, HST-5, and the determination of total protein concentration

The concentration of chosen antimicrobial peptides was determined by the competitive enzyme-linked immunosorbent assay (ELISA). About 50 μl of standard β-defensin-2, HST-5, or diluted saliva sample (saliva was diluted to 1:100 dilution by adding 4 μl of saliva to 400 μl of phosphate buffered saline), 50 µl of enzyme (horseradish peroxidase)-labeled antigen, and 50 µl of anti-β-defensin-2, anti-HST-5 monoclonal antibodies (1:800) were added to the secondary antibody-coated 96-well microliter plates and reacted at room temperature for 30 min. Then, the solution was decanted, and 300 µl of washing solution was added to each well and also decanted; this procedure was repeated three times. Then, 100 µl of *o*-phenylenediamine (OPD) solution was added. After the enzyme reaction at room temperature for 15 min, the reaction was stopped by adding 100 µl of 0.25 mol l^−1^ sulfuric acid. A standard curve was performed with each assay using HBD-2 and HST-5 at concentrations of 0.02, 0.05, 0.1, 0.5, 1, and 2 µg. Briefly, the wells of high-binding 96-well microliter plates (Corning Incorporated, Big Flats, NY, USA) were coated with 50 µl of each β-defensin-2, HST-5 concentration, or TBS in the control wells. The optical density (OD) was measured at 450 nm [29], and the examined protein concentration in saliva was expressed as microgram per 1 ml saliva (µg ml)^−1^. Salivary protein level was measured by using a standard protein assay kit (BIO-RAD, Hercules, CA, USA) with bovine serum albumin as a standard. Three samples were collected from each patient on three different occasions under same conditions. The experiments were performed in duplicates in all three occasions.

### Statistical analysis

The mean values were compared using the unpaired *t* test and one-way ANOVA with Scheffe’s multiple comparison test, respectively. Linear regression analysis was used to evaluate the simple correlation between examined protein level and *Streptococcus mutans*/*Lactobacillus rhamnosus*/*Streptococcus sanguinis*/*Streptococcus mitis* CFU counts. Multiple linear regression analysis was performed to determine the independent contribution of explanatory variables to bacterial strain CFU counts using age, sex, β-defensin-2, and HST-5 concentration.

## Results

Salivary HST-5 and β-defensin-2 levels in patients with ECC (50.75 ± 2.11; 2.29 ± 0.05 ng/ml, respectively) were significantly increased compared with control (healthy subject) (15.29 ± 1.16; 2.15 ± 0.07 ng/ml, respectively) (*p* < 0.001, and 0.04, respectively; Table 1).

When the results were analyzed according to the caries severity score, salivary HST-5 and β-defensin-2 levels were significantly higher in severe group (cavitated lesion) which was considered to be ≥3 in ICDAS II codes (53.06 and 2.30 ng/ml, respectively) compared to the mild group (1–2 in ICDAS II codes) with the initial demineralization involvement (white spots/non-cavitated lesion) (44.53 and 2.22 ng/ml, respectively) (*p* = 0.030 and *p* = 0.021, respectively).

Cariogenic profile was the only bacterial species recovered in severe and mild group of patients.

**Fig. 3:**
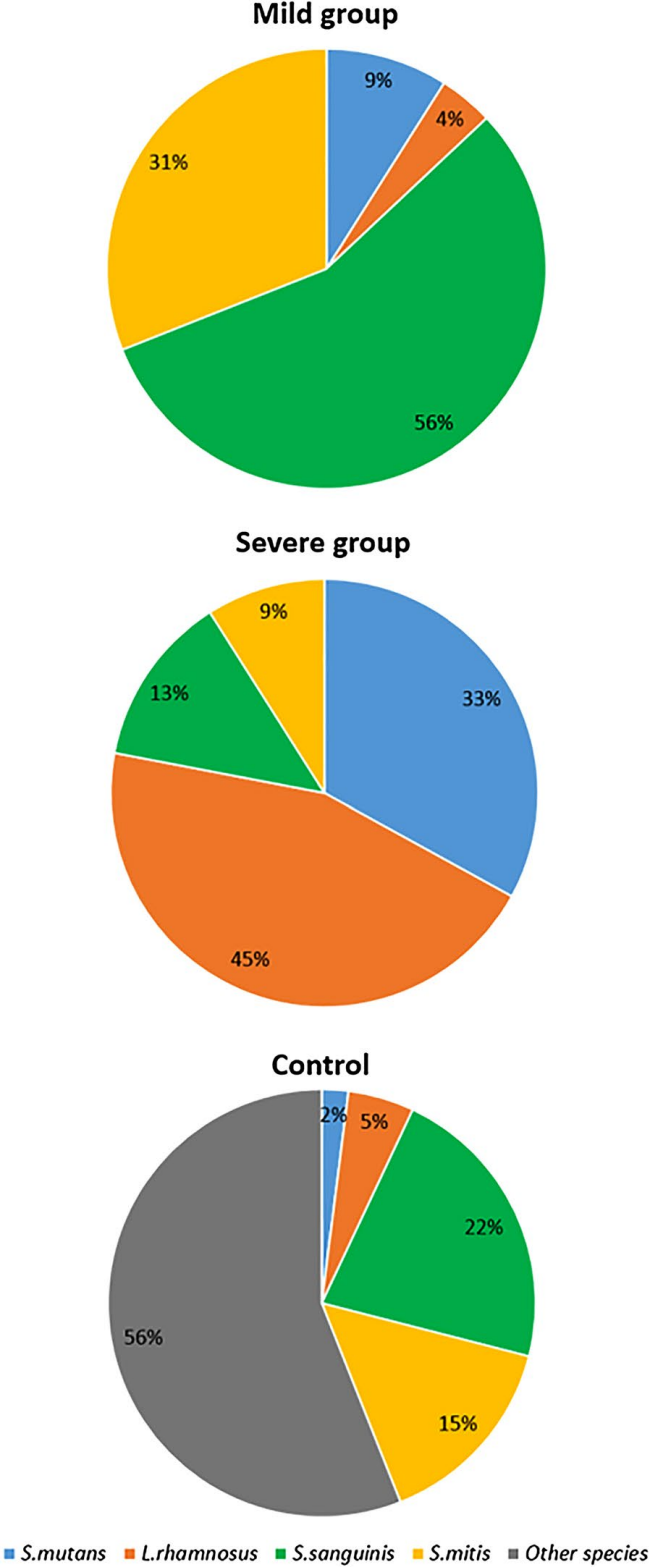
Cariogenic profile in early childhood caries between the severe, the mild, and control groups

Noticeable is the high proportion of *S. mutans* and *L. rhamnosus* with severe group in relation to mild and control groups. Bacterial cultures demonstrated significant differences in *Streptococcus mutans*/*Lactobacillus rhamnosus*/*Streptococcus sanguinis*/*Streptococcus mitis* profiles between the severe group (dentine damage), the mild group (white spots), and control group (*p* = 0.04, Fig. 3).

Alternatively, ELISA results demonstrated a significant growth in HST-5 (*p* = 0.03) and β-defensin-2 (*p* = 0.04) concentrations in the saliva of the severe group compared with the mild group, where HST-5 levels in the mild population ranged between 40.11 and 45.23 ng/ml (average 42.55 ± 0.36 ng/ml) and in severe group (dentine damage) between 49.37 and 51.86 ng/ml (average 50.55 ± 0.50 ng/ml) (Table 2).

**Table 2:** Salivary HST-5 and β-defensin-2 levels in severe and mild groups of patients

	The severe group (*n* = 24)		The mild group (*n* = 17)		p*
	Mean (ng/ml)	SD	Mean (ng/ml)	SD	
HST-5	50.55	0.50	42.55	0.36	0.03
β-defensin-2	2.22	0.19	1.74	0.21	0.04

No correlation was observed between HST-5, β-defensin-2 levels, and age, gender, social habits, diets, medications or any other co-founding factors (not published results)

## Discussion

Dental caries is an infectious disease and is one of the most common diseases among children and teenagers in the world [30]. Despite numerous studies on etiology and development of the disease, scientists still do not agree on some of its aspects. Until recently, *S. mutans*, which has the ability to form biofilm in the oral cavity, was considered to be the main etiological factor of caries [31, 32]. The one which is currently prevailing is the opinion on complex bacterial component of caries etiology. This process, apart from *S. mutans*, involves several other key bacterial species, i.e.: *S. sobrinus*, *Lactobacillus acidophilus*, and *Actinomyces viscosus* [33–37].

The objective of the study was to determine the levels of HST-5, β-defensin-2 and to compare them with bacterial profiles depending on caries progression. The obtained results demonstrated significantly higher HST-5 and β-defensin-2 levels in saliva of children with severe ECC compared to patients with the initial demineralization involvement (white spots/non-cavitated lesion).

The obtained results are consistent with the results of Gornowicz et al., who demonstrated increased level of HST-5 in group of children with severe caries, but higher concentrations were observed in children with severe form of the disease compared to patients with the initial demineralization involvement [38].

Increased activity of HST-5 was observed in wide pH range. However, in low pH (with presence of caries dental changes) protonation of histidine residues occurs, additionally increasing the antimicrobial force [18, 39]. Not only the primary form of HST-5 but also its synthetic equivalent, P-113 peptide, maintains their biological potential. Due to the proteolytic degradation of HST-5 in the oral cavity, P-113 exhibits significantly greater effectiveness toward cariogenic strains of *S. mutans*. This peptide penetrates into the cellular cytoplasm without disturbing the cell membrane integrity and has a significant affinity to microbial DNA [40–42].

Considering the relationship of β-defensin-2 (HBD-2) to caries, Ribeiro et al., Tao et al., and Dale et al. obtained the results that were inconsistent with the results presented in this study [43–45]. According to these authors, HBD-2 does not correlate with the occurrence of caries which is in contrast with HBD-1 and HNP-3 (α-defensin-3).

Also, Phattarataratip and coworkers did not demonstrate the relationship of HBD-2 with the symptoms of the disease; however, a trend in the increase of defensin level together with the increase of total content of bacteria from the *mutans streptococci* group was observed. Strains of *S. mutans* isolated from persons with caries had greater resistance to β-defensin-2 compared to strains isolated from control group [46].

It is generally known that HBD-2 exhibits the greatest activity toward Gram-negative bacteria and to a lower level toward Gram-positive bacteria. Cariogenic species, which are the most sensitive to their activity, are *S. mutans* and *S. sobrinus* [47], although HBD-2 are, compared to other salivary proteins, for instance, cathelicidin (LL37) significantly more specific toward *S. mutans*, which was corroborated by the results obtained in other studies [48].

This study demonstrated that the amount of specific salivary proteins of the host may influence colonization of each species of bacteria, including *S. mutans* and also that domination of strains considered to be more cariogenic, such as *S. mutans* is closely linked to the level of caries advancement (severe and moderate caries) which is reflected in increased level of selected salivary proteins of the host, both HST-5 and β-defensin-2.

When searching for the relation between the levels of HST-5 and β-defensin-2 and the number of cariogenic species of bacteria, the occurrence of specific bacterial profiles depending on the levels of the determined proteins was noticed. These data correlated with clinical data on caries severity. A recent study evaluating population of oral cavity microbes in children 3–12 years of age suggests that teeth microflora is related to an entire ecosystem of species of bacteria that it is made up of and not only a small portion related to a small number of selected, specific pathogenic species of bacteria, which together determine caries development [49, 50]. The results which were obtained from the group of children with mild caries often being described as white spots (*Streptococcus mutans*/*Lactobacillus rhamnosus*/*Streptococcus sanguinis*/*Streptococcus mitis* 9 %–4 %–56 %–31 %) are consistent with results of Aas et al. [51] and van Houte et al., who demonstrated that 10 % of children and teenagers with caries (2–21 years of age) do not have *S. mutans* at all. The participation of other bacterial species in the development and progression of dental caries is suggested, such as Lactobacillus, Veillonella, Bifidobacterium, Propionibacterium, acidogenic species independent of *Streptococcus mutans* (i.e.: *S. gordonii*, *S. oralis*, *S. mitis*, *S. anginosus*) [52], *Actinomyces*, and *Atopobium*, which was confirmed in this study. In the case of the group of the so-called white spots, the participation of *S. mutans* was higher compared to the control group (9 % white spot >2 % control), which was confirmed in the study by van Houte et al. in which *S. mutans* constituted 0.001 to 10 % [53]. Species of Streptococcus (apart from *S. mutans*) and Actinomyces are considered the main culprits of enamel damage. In the case of *S. mutans* and Lactobacillus absence, the initial demineralization of enamel may be induced by early colonizers (*S. sanguinis*, *S. mitis*, and *S. oralis*), which was also presented in this study, where *Streptococcus sanguinis* constituted 56 % in children of the white spot group.

In children from severe group participation of *S. mutans* amounted to 33 %, which is consistent with the results of Gross et al. [54] and Sampaio-Maia and Monteiro-Silva [55] in which in dentine damage, the participation of *S. mutans* amounted to approximately 30 % of total microflora. *S. mutans* was not a dominant species in the advanced stage of caries, where *Lactobacillus*, *Bifidobacterium*, and *Prevotella* dominated. Virulence of populations of cariogenic bacteria correlates with the accepted phenotype for a given environment related to bacteria acidogenic potential which may induce changes of the environment leading to the development of dental caries.

Under physiological conditions, the oral cavity of children compared to adult persons has higher percentage of bacteria belonging to phyla: Firmicutes (genera Streptococcus, Veillonella, Lactobacillus, and Granulicatella) and Actinobacteria (genera Rothia and Actinomyces) and lower percentage of bacteria from phyla Bacteroidetes (genera Bacteroidales and Prevotella), Fusobacteria (genus Fusobacterium), Spirochaetes, and TM7 strain [56]. Interestingly, percentage of cariogenic bacteria increases with the age of children. This change consists in a shift from populations of aerobic bacteria or facultative Gram-positive cocci to relatively anaerobic Gram-negative bacteria [50]. In this study, in the control group, non-pathogenic species dominated (*Streptococcus mutans*/*Lactobacillus rhamnosus*/*Streptococcus sanguinis*/*Streptococcus mitis* 2 %–5 %–22 %–15 %–56 % other species), which confirms the hypothesis on environment-dependent bacterial profile.

This study indicates great opportunities of using HST-5 in combination with β-defensin-2 in dentistry. If the potential of antibacterial peptides is to be utilized as prospective biomarkers of various diseases, including development and progression of dental caries together with proper hygiene of the oral cavity, patient education and professional prevention measures, a new approach will be created, leading to improved prevention, diagnostics, and dental caries treatment.

Antimicrobial peptides, to which mentioned proteins belong, have numerous favorable characteristics such as wide range of activity, quick antibacterial effect, competitiveness toward traditional antibiotics, thanks to which they are potential tools for the treatment of numerous humans and animal diseases caused by biological factors.

The results of the above-described study, in which we observed an increase in the level of histatin-5 and β-defensin-2 along with the severity of dental caries, prove that selected antimicrobial peptides may serve as a tool in the prevention and treatment of dental caries especially in children that are at risk of this disease. Furthermore, the overall demand for the use of natural substances cause the increased interesting in the use of antimicrobial peptides. Consequently, there are possibilities to develop new strategies to treat this disease. Antibacterial peptides are the alternatives to conventional antibiotic treatment. Many studies have been performed on hundreds of antimicrobial peptides that can be used for a more targeted approach associated with selective control or elimination of strains causing tooth decay in children.

In addition, antimicrobial peptides properties are much safer for the youngest users. Our studies indicate that antimicrobial peptides such as histatin-5 and β-defensin-2 should be further studied. Their potential use in oral therapy should be also discussed.

## Conclusions

This study has shown that there is a relationship between the amount of salivary β-defensin-2 and Histatin-5 and caries advancement (severe and moderate caries). The occurrence of specific bacterial profiles depending on the levels of the determined proteins was noticed. The use of the saliva proteins concentration could have potential applications in the monitoring of oral diseases, such as caries and periodontal diseases.

## Abbreviations

AEP: acquired enamel pellicle
AMPs: antimicrobial peptides
ATP: adenosine triphosphate
CCR6: C–C chemokine receptor type 6
CFU: colony-forming unit
DMF: decayed, missing, filled index
DNA: deoxyribonucleic acid
ECC: early childhood caries
EGF: epidermal growth factor
ELISA: enzyme-linked immunosorbent assays
ERK: extracellular-signal-regulated kinase
HAdV: human adenowirus
HBD: human β-defensin
HIV-1: human immunodeficiency virus type 1
HLR-S: modified medium of Ritz
HNP: human neutrophil peptide
HST: histatin
HSV-1: herpes simplex virus type 1
ICDAS: International Caries Detection and Assessment System
IFN-γ: interferon γ
IκBα: nuclear factor of κ light polypeptide gene enhancer in B-cells inhibitor, α
IKK: IκB kinase
IL: interleukin
JNK: c-Jun N-terminal kinase
LBP: lipopolysaccharide binding protein
LPO: lactoperoxidase
LPS: lipopolysaccharide
TLR: toll-like receptor
MAPK: mitogen-activated protein kinase
MEK: mitogen-activated protein kinase
MMP: matrix metalloproteinases
NFκB: nuclear factor κ-light-chain-enhancer of activated B cells
OD: optical density
OPD: *o*-phenyl-enediamine
Raf: proto-oncogene serine/threonine-protein kinase
ROS: reactive oxygen species
sCD14: soluble CD14
sIgA: secretory immunoglobulin A
Src: proto-oncogene tyrosine-protein kinase Src
TBS: tert-butyldimethylsilyl ether
TNF-α: tumor necrosis factor α
